# Haemolytic anaemia in an HIV-infected patient with severe *falciparum *malaria after treatment with oral artemether-lumefantrine

**DOI:** 10.1186/1475-2875-11-91

**Published:** 2012-03-27

**Authors:** Angela Corpolongo, Pasquale De Nardo, Piero Ghirga, Elisa Gentilotti, Rita Bellagamba, Chiara Tommasi, Maria Grazia Paglia, Emanuele Nicastri, Pasquale Narciso

**Affiliations:** 1 National Institute for Infectious Diseases "Lazzaro Spallanzani", Via Portuense 292, 00149 Rome, Italy

**Keywords:** Severe malaria, Artemisinin-based combination therapy (ACT), Haemolytic anaemia, Drugs and haemolytic anaemia

## Abstract

Intravenous (i.v.) artesunate is now the recommended first-line treatment of severe *falciparum *malaria in adults and children by WHO guidelines. Nevertheless, several cases of haemolytic anaemia due to i.v. artesunate treatment have been reported. This paper describes the case of an HIV-infected patient with severe *falciparum *malaria who was diagnosed with haemolytic anaemia after treatment with oral artemether-lumefantrine.

The patient presented with fever, headache, and arthromyalgia after returning from Central African Republic where he had been working. The blood examination revealed acute renal failure, thrombocytopaenia and hypoxia. Blood for malaria parasites indicated hyperparasitaemia (6%) and *Plasmodium falciparum *infection was confirmed by nested-PCR. Severe malaria according to the laboratory WHO criteria was diagnosed. A treatment with quinine and doxycycline for the first 12 hours was initially administered, followed by arthemeter/lumefantrine (Riamet^®^) for a further three days. At day 10, a diagnosis of severe haemolytic anaemia was made (Hb 6.9 g/dl, LDH 2071 U/l). Hereditary and autoimmune disorders and other infections were excluded through bone marrow aspiration, total body TC scan and a wide panel of molecular and serologic assays. The patient was treated by transfusion of six units of packed blood red cell. He was discharged after complete remission at day 25. At present, the patient is in a good clinical condition and there is no evidence of haemolytic anaemia recurrence.

This is the first report of haemolytic anaemia probably associated with oral artemether/lumefantrine. Further research is warranted to better define the adverse events occurring during combination therapy with artemisinin derivatives.

## Background

Artemisinins were discovered in 1971 from a herb, *Artemisia annua*, known in the past for their activity against intermittent fever [1]. Now, artemisinin and its derivatives have become essential components of artemisinin-based combination therapy (ACT) [2]. Intravenous (i.v.) artesunate is the recommended first-line treatment of severe falciparum malaria in adults and children, including cerebral malaria, because they produce rapid parasite/fever clearance, show fewer adverse effects, and have not been associated with occurrence of less resistance [3,4]. The World Health Organization's (WHO) up-to-date guidelines recommend artesunate as the treatment of choice for adults and children with severe malaria [5]. However, quinine is still the primary treatment for severe non-multidrug resistant *Plasmodium falciparum *malaria in Europe because i.v. artesunate is not registred for this indication. This paper describes the occurrence of a case of haemolytic anaemia in an HIV-infected patient with *falciparum *malaria treated with intravenous (i.v.) quinine and doxycycline for the first 12 hours and then with artemether-lumefantrine

## Case report

A 34-year-old man presented with a seven-day history of fever, headache, and arthromyalgia after returning from Central African Republic. He is Italian and he had been working for governmental and non-governmental organizations for several years. His past medical history included HIV infection known since October 2010 with no anti-retroviral treatment. At admission on 18 July, 2011, the physical examination revealed prostration, severe obesity (BMI 42), pallor, shallow tachypnoic breathing, and hepato-splenomegaly. Blood examinations showed acute renal failure (creatinine 3.29 mg/dl), anaemia (Hb 7.4 g/dl), thrombocytopaenia (PLT 17000), glucose 88 mg/dl, AST 108 U/l, ALT 33 U/l, bilirubin total 2.9 mg/dl, bilirubin direct 1.5 mg/dl, albumin 2,5 g/dl, G6PDH 16 U/gHb, hypoxia and metabolic acidosis (pH 7.35, pO2 62 mmHg, HCO3^- ^16.5 mmol/l) (see Table 1). Polymerase Chain Reaction (PCR) and light microscopy on peripheral blood smear detected *P. falciparum *with a 6% parasitaemia. Severe malaria, according to WHO criteria (hyperparasitaemia, metabolic acidosis, renal impairment), was diagnosed [5].

**Table 1:** Laboratory data

Test, unit	Admission 18 July	Day 7 25 July	Day 10 28 July	Day 17	Day 25 12 Aug	Normal values
Glucose, g/dl	88		0.97			70-1.10

Urea, g/l	2.16		0.33			0.10-0.50

Creatinine, mg/dl	3.29		0.65			0.5-1.4

Potassium, mEq/l	3.5		4.9			3.6-5.5

Bilirubin total/direct, mg/dl	2.9/1.5	1.4/0.7	1.6/0.6	1.9/0.8		< 1.2/0.3

AST/ALT, U/l	108/33	32/26	40/23			< 40

**Erythrocytes**, 10^6^/mm3	2.43	2.6	2.16	1.81	2.91	3.8-6.1

Haemoglobin, g/dl	7.4	8	6.9	5.9	9.6	12-18

Haematocrit, %	22	24	20	18	29	36-52

Reticulocytes %			4.8	8.2		0.5-2.0

Platelets, 10^3^/mmc	17	114	127	98	152	80-400

White blood cells, 10^3^/mmc	5.1	6.7	5.3	2.6	3.5	4.3-10.8

Neutrophils %	75.5	41.1	23.1	31.6	36.6	40-75

Lymphocytes %	20	42.7	62	49	41.2	20-51

CD4 cells/mmc (%)		569 (23.2)				(36-44)

HIV-RNA, cp/ml	161769					

Erythrocyte Sedimentation Rate, mm/hr	122		> 140	> 140		< 15

C-Reactive proteine mg/dl	21			4.7		< 0.6

Thick and thin smears	Pos	Neg, day 3	Neg			

PCR for *Plasmodium falciparum*	Pos	Neg, day 3	Neg			

Parasitaemia	6%					

Coombs test direct/indirect			neg/neg	neg/neg		

The patient was initially treated with i.v. quinine loading dose (20 mg/kg, 1500 mg) in combination with oral doxycycline (100 mg) for the first 12 hours followed by oral fixed-dose co-formulated artemether/lumefantrine (Riamet^®^) for a further three days. At admission, the blood test detected moderate-severe anaemia (Hb 7.4 g/dl and HCT 22%) and a transfusion with one unit of packed red blood cells (RBC) was prescribed. Because of the persistence of the anaemia, the patient was transfused with a further RBC transfusion on day 4 (Hb 6.7 g/dl and HCT 20%). Parasite clearance time was 72 hours. Renal and pulmonary functions immediately improved but fever was still reported up to 28 July, when the diagnosis of severe haemolytic anaemia was performed (Hb 6.9 g/dl, haptoglobin < 7.19 mg/dl, reticulocytes 4.8%, LDH 2071 U/l). The patient was transfused with an additional four packed blood transfusions on two different days.

Direct and indirect anti-globulin tests, cold agglutinin test, and auto-immune assays were all negative; PCR and antibody detection for chikungunya, dengue, serology for *B19 Parvovirus, Leishmania *species, *Mycoplasma pneumoniae, Chlamydia pneumoniae, Mycobacterium tuberculosis *complex, atypical mycobacteria, *Salmonella typhi *and *paratyphi, Trypanosoma *spp., *Toxoplasma gondii*, and *Treponema pallidum *were all negative; antigens of *Cryptococcus neoformans *were not detectable; PCR and peripheral blood smears for *Plasmodium *species were persistently negative. Bone marrow aspiration was negative for infectious agents and lympho-proliferative diseases. A total body CT scan reported splenomegaly. After significant improvement of his clinical condition, the patient was discharged on 12 August (Hb 9.6 g/dl). Five weeks after discharge, haemoglobin, haematocrit, reticulocytes count were all within normal values (see Table 1). The patient is now in good clinical condition and back to his daily working life with no evidence of haemolytic anaemia recurrence.

Written informed consent was obtained from the patient for publication of this case report.

## Discussion

There are multiple causes of haemolytic anaemia, and the clinical presentation can differ depending on the etiology. Haemolysis may be extravascular due to autoimmune disorders or to hereditary spherocytosis, intravascular (e.g. associated to insertion of prosthetic cardiac valves, G6PDH deficiency, thrombotic thrombocytopenic purpura or disseminated intravascular coagulation), or intramedullary-related due to pernicious anaemia and thalassemia major. The haemolytic disorder can be hereditary as in case of G6PDH deficiency [6], haemoglobinopathies and RBC membrane abnormalities, or acquired, as in the case of immune disorders [7], exposure to toxic chemicals and drugs [8], paroxysmal nocturnal haemoglobinuria [9], traumatic and microangiopathic haemolysis, hypersplenism or infections [10].

In this report, hereditary and auto-immune disorders were excluded. Infectious agents other than malaria were searched for with a wide panel of molecular and serologic assays and through bone marrow examination. All the results were negative. CT scan and bone marrow analysis excluded lymphoproliferative disorders. Malaria was treated with i.v. quinine and doxycycline for the first 12 hours only, followed by Riamet^® ^for 72 hours. At day 10 a diagnosis of severe haemolytic anaemia was established and it was treated by transfusion with six units of packed red blood cells. The duration of haemolysis was 15 days. The patient was discharged on 12 August after complete remission from severe malaria and haemolytic anaemia.

There are few cases described in literature of haemolytic anaemia during or after treatment with i.v. artesunate alone or combined with mefloquine. Yasouka *et al *reported the case of a Nigerian male with severe *P. falciparum *malaria initially treated with mefloquine. After one day of treatment, because of the worsening clinical condition of the patient and the increase of the parasitaemia, therapy with i.v. artesunate was initiated. Parasite clearance was obtained within 20 hours after the first administration of artesunate, but fever persisted for a further seven days and haemolytic anaemia was observed, requiring blood transfusion [11]. Yoshizawa *et al *described the case of a young woman with *P. falciparum *malaria who was successfully treated with i.v. artesunate, but showed worsening anaemia after artesunate administration [12]. Itoda *et al *observed severe haemolytic anaemia and jaundice on day 11 after i.v. artesunate administration in a 68-year-old Japanese woman affected by severe malaria [13]. Recently, Zoller *et al *reported a series of 25 travellers with severe malaria treated with i.v. artesunate. Among them, six patients developed haemolytic anaemia week after treatment possibily related to artesunate or had persistent signs of haemolytic activity until six weeks after the first dose of i.v. artesunate [14]. In this case series, patients with post-treatment haemolysis had received a higher cumulative dose of i.v. artesunate and were treated for longer periods.

This appears to the first reported case of haemolytic anaemia after treatment with oral fixed-dose co-formulated artemether/lumefantrine. The observation supports the hypothesis that haemolysis might occur as a consequence of artemether treatment. Efficacy and safety profiles of i.v. artesunate and ACT should be prospectively evaluated, and patients should be monitored for signs of haemolysis after parasitological cure.

## Competing interests

The authors declare that they have no competing interests.

## Authors' contributions

AC, PDN, RB, EN collected and collated case information and details and obtained consent. All authors participated in drafting the medical part of the manuscript. MGP performed the PCR amplification and immunohistochemistry. PG and EG were involved in literature search. All authors read and approved the final manuscript.
